# DNA Polymerase B1 Binding Protein 1 Is Important for DNA Repair by Holoenzyme PolB1 in the Extremely Thermophilic Crenarchaeon *Sulfolobus acidocaldarius*

**DOI:** 10.3390/microorganisms9020439

**Published:** 2021-02-20

**Authors:** Hiroka Miyabayashi, Hiroyuki D. Sakai, Norio Kurosawa

**Affiliations:** 1 Department of Environmental Engineering for Symbiosis, Graduate School of Science and Engineering, Soka University, 1-236 Tangi-machi, Hachioji, Tokyo 192-8577, Japan; e19m5707@soka-u.jp; 2 Department of Science and Engineering for Sustainable Innovation, Faculty of Science and Engineering, Soka University, 1-236 Tangi-machi, Hachioji, Tokyo 192-8577, Japan; shiroyuki@soka.ac.jp

**Keywords:** hyperthermophilic archaea, *Sulfolobus acidocaldarius*, DNA polymerase B1, DNA replication, PolB1-binding protein

## Abstract

DNA polymerase B1 (PolB1) is a member of the B-family DNA polymerase family and is a replicative DNA polymerase in Crenarchaea. PolB1 is responsible for the DNA replication of both the leading and lagging strands in the thermophilic crenarchaeon *Sulfolobus acidocaldarius*. Recently, two subunits, PolB1-binding protein (PBP)1 and PBP2, were identified in *Saccharolobus solfataricus*. Previous in vitro studies suggested that PBP1 and PBP2 influence the core activity of apoenzyme PolB1 (apo-PolB1). PBP1 contains a C-terminal acidic tail and modulates the strand-displacement synthesis activity of PolB1 during the synthesis of Okazaki fragments. PBP2 modestly enhances the DNA polymerase activity of apo-PolB1. These subunits are present in *Sulfolobales*, *Acidilobales*, and *Desulfurococcales*, which belong to Crenarchaea. However, it has not been determined whether these subunits are essential for the activity of apo-PolB1. In this study, we constructed a *pbp1* deletion strain in *S. acidocaldarius* and characterized its phenotypes. However, a *pbp2* deletion strain was not obtained, indicating that PBP2 is essential for replication by holoenzyme PolB1. A *pbp1* deletion strain was sensitive to various types of DNA damage and exhibited an increased mutation rate, suggesting that PBP1 contribute to the repair or tolerance of DNA damage by holoenzyme PolB1. The results of our study suggest that PBP1 is important for DNA repair by holoenzyme PolB1 in *S. acidocaldarius*.

## 1. Introduction

DNA polymerases (DNAPs) are enzymes that synthesize DNA, playing a central role in DNA replication and repair. Accurate and timely replication is important for all living organisms. In general, replicative DNAPs are highly processive, accurate, and exhibit 3′ to 5′ exonuclease activity [1]. DNA damage is largely unavoidable, and efficient repair of that is important for accurate DNA replication [2,3]. Generally, non-replicative DNAPs are responsible for various and often short-length DNA synthesis in repair. In bacteria, a C-family polymerase, namely, PolIII, synthesizes the leading and lagging strands. PolIII consists of a ten-component complex: The catalytic part (α-, ε-, and θ-subunits), the clamp loader or γ-complex (γ-, δ-, δ’-, ζ-, χ-, and ψ-subunits), and the sliding clamp (β2) [4,5]. In Eukarya, two B-family polymerases, Polε and Polδ, replicate the leading and lagging strands, respectively [6,7]. These DNAPs are multi-subunit proteins containing a catalytic subunit, a regulatory subunit, and an assortment of accessory subunits [5,8,9]. Most archaea except for Crenarchaea possess a D-family polymerase and at least one B-family polymerase [7,10,11]. The B-family polymerase PolB3 is distributed widely in almost all archaea except Thaumarchaota [10,11]. The euryarchaea *Methanococcus maripaludis* and *Thermococcus kodakarensis* have PolD and PolB3. In genetic studies of these species, *polD* is essential for viability, but *polB3* is not; that is, PolD replicates both the leading and lagging strands [12,13,14]. Crenarchaea lack PolD, but possess at least two B-family polymerases [7,10,11]. The extremely thermophilic crenarchaeon *Sulfolobus acidocaldarius* has four DNAPs: PolB1, PolB2, PolB3, and Dbh. Previous in vivo experiments indicated that PolB1 is a replicative polymerase for both leading and lagging strands since the triple gene-deletion strains lacking *polB2*, *polB3*, and *dbh* had been successfully isolated [15]. In short, it is plausible that PolD in Euryarchaea and PolB1 in Crenarchaea are replicative DNAPs [11,12,14,15,16].

PolD is composed of a large catalytic subunit (DP2) and a smaller subunit with 3′ to 5′ exonuclease activity (DP1) [17,18]. On the other hand, PolB1 has been believed to be a single-subunit enzyme since the characterization of PolB1 in *Sulfolobus acidocaldarius* in 1985 [19]. In 2017, two subunits, PolB1-binding protein (PBP)1 and PBP2, were identified in *Saccharolobus solfataricus* [20,21]. PolB1 was revealed to be a multi-subunit protein. PBP1 and PBP2 influence the core activity of apoenzyme PolB1 (apo-PolB1) [20]. PBP1 contains a C-terminal acidic tail and modulates the strand-displacement synthesis activity of PolB1 during the synthesis of Okazaki fragments [20]. Thus, PBP1 limits the needless elimination and resynthesis of DNA in the preceding Okazaki fragment for efficient lagging strand DNA synthesis [20]. PBP2 modestly increases the DNA polymerase activity of apo-PolB1 [20,22]. In addition, it reduces the inhibition of DNA synthesis by PBP1 [20,22]. These subunits are present in *Sulfolobales*, *Acidilobales*, and *Desulfurococcales*, which belong to Crenarchaea. However, it has not been determined whether these subunits are essential for the activity of apo-PolB1.

To examine whether these subunits are essential for the activity of apo-PolB1 in *S. acidocaldarius*, we attempted to construct strains completely lacking the *pbp1* and *pbp2* genes and characterized their mutant phenotypes, examining sensitivity to numerous types of DNA damage (i.e., UV irradiation, DNA-damaging agents, heat shock, and DNA replication inhibitors) and mutation rates. We report that holoenzyme PolB1 (apo-PolB1 with PBP1 and PBP2) is responsible for the repair of most DNA damage in addition to DNA replication in *S. acidocaldarius*.

## 2. Materials and Methods

### 2.1. Strains and Growth Conditions

The growth conditions were previously reported [23]. *S. acidocaldarius* strain DP-1 (Δ*pyrE* Δ*suaI* Δ*phr*), which is pyrimidine-auxotrophic, restriction endonuclease *Sua*I-deficient and DNA photolyase Phr-deficient was used as the parent strain [23,24] for construction of strain HM-8 (Table 1). These strains were cultivated in the xylose and tryptone (XT) medium (pH 3) [15,25] at 75 °C with or without shaking (160 rpm). For plate medium, identical components of 1× basal salts containing 2.9 g MgSO_4_·7 H_2_O and 0.5 g CaCl_2_·2H_2_O, and Gellan Gum (0.65 g/L) were used. Uracil (0.02 g/L) was added to XT medium (XTU) for cultivation of pyrimidine-auxotrophic strain. 5-fluoro-orotic acid (FOA) (50 μg/mL) was added to the XTU medium (XTUF) for counter selection in the pop-out recombination (Section 2.3) and for spontaneous mutation analysis (Section 2.7).

### 2.2. General DNA Manipulation

The reagents used in these experiments were prepared as previously described [23]. EmeraldAmp MAX PCR Master Mix (Takara Bio, Kusatsu, Shiga, Japan) was used for PCR amplification. PCR products were purified using the NucleoSpin Gel and PCR Clean-up kit (Macherey-Nagel, Düren, Germany). The Sanger sequencing was performed by the Eurofins Genomics (Tokyo, Japan, https://www.eurofinsgenomics.jp/).

### 2.3. Construction of the PolB1-binding Protein Gene-Deleted Strains

The multiple gene knockout strategy with one-step PCR (MONSTER) was used to prepare *pbp1* (Saci_0746) and *pbp2* (Saci_1566) knockout cassettes (MONSTER-pbp1 and MONSTER-pbp2, respectively) and to construct *pbp1* and *pbp2* deletion strains [23]. In addition, another *pbp2* knockout cassette (MONSTER-pbp2n) was prepared to delete *pbp2* in different deletion regions. The DNAs and PCR primers used in this study are listed in Table 1 and Table 2, respectively. In brief, the MONSTER-pbp1 cassette was amplified from placSpyrE as a template using the MONSTER-pbp1-F/R primers. Similarly, the MONSTER-pbp2 and MONSTER-pbp2n cassettes were amplified using MONSTER-pbp2-F/R primers and MONSTER-pbp2n-F/R primers, respectively. The purified PCR products (100–200 ng/μL in 5 mM Tris-HCl, pH 8.5) were used for subsequent electrotransformation.

The transformation procedure has been previously described in detail [23]. To delete *pbp1*, 2 μg of MONSTER-pbp1 was electroporated (15 kV/cm, 9 ms) into 200 μL of DP-1 competent cells harvested at the mid-log phase (the optical density of the culture at 600 nm (OD_600]_ = 0.34–0.43) in a 2 mm electroporation cuvette (Bio-Rad, Hercules, CA, USA). Similarly, MONSTER-pbp2 or MONSTER-pbp2n was electroporated into 200 μL of DP-1 competent cells to delete *pbp2*. After electroporation, the sample was spread onto an XT plate. After five days of cultivation at 75 °C, blue transformant colonies were selected by spraying a 10 mg/mL 5-bromo-4-chloro-3-indolyl-β-d-galactopyranoside (X-gal) solution in 40% *N*,*N*-dimethylformamide (DMF) diluted with 0.85% NaCl solution on the plate, followed by incubation at 75 °C for one day. The genotypes were confirmed using the outer primers (pbp1-out-F/R). Single-colony isolation followed by genotypic analysis using the outer primers was performed at each step for the selection of intermediates and gene deletion strains. To exclude translocation of the *pbp1* gene in any genomic locus of the *pbp1*-deleted strain HM-8, PCR analysis was performed using inner primers pbp1-in-F/R, which anneal with the inner (deleted) region of the *pbp1* gene.

The deletion of the *pbp1* gene was also checked by sequencing analysis. A *pbp1* gene was amplified from cultures of DP-1 and HM-8 using the outer primers (pbp1-out-F/R). Each *pbp1* gene was sequenced by Sanger method using the outer primer (pbp1-out-F) (Table 2).

### 2.4. Construction of the pyrE-Proficient Strains

The *pyrE*-proficient strain was constructed as previously described [26]. A short cassette carrying 18 bp-deletion of *pyrE* gene sequence of the pyrimidine-auxotrophic strain MR31 [27], and 150 bp and 101 bp of the 5′ and 3′ flanking regions, respectively, was amplified from the *S. acidocaldarius* DSM639 genomic DNA using SAMR31-F/R primers under the following conditions: 94 °C for 3 min; 30 cycles of 94 °C for 30 s, 58 °C for 30 s, and 72 °C for 30 s; and a final extension at 72 °C for 3 min. The purified PCR products were electroporated (15 kV/cm, 9 ms) into 200 μL of competent cells of the pyrimidine-auxotrophic strains DP-1 or HM-8 harvested at early to mid-log phase (OD_600_ = 0.34 and 0.30, respectively), and the resulting colonies were isolated. After a second single-colony isolation, the manipulated regions of genomic DNA of each strain were checked by PCR using SAMR31-F/R primers. The strain containing the expected lengthening of this interval was used as the *pyrE*-proficient strain.

### 2.5. Growth Temperature Range

For characterization of the growth temperature range, overnight cultures of DP-1 and HM-8 (late-log to stationary phase) were inoculated into 6 mL of XTU liquid medium to yield an initial OD_600_ = 0.005 in triplicate. The cells in loosely capped glass tubes were cultivated at 50–80 °C (temperature range from minimal to maximal growth temperature) with intervals of 5 °C without shaking on the block heater.

### 2.6. DNA Damage Sensitivity Tests

The sensitivity tests to the UV irradiation or DNA-damaging agents of the mutant and parental strains were performed by using the exact same protocol as previously described [23]. The survivability test after exposure of DNA damaging agents was also performed as described in the same literature except the plates were incubated at 75 °C for 6 days.

### 2.7. Spontaneous Mutation Analysis

The rates of mutations that inactivate the *pyrE* gene were determined by previously described methods [26]. The overnight culture of the *pyrE*-proficient strains of DP-1 (OD_600_ = 0.67–0.69, 1.54–1.88 × 10^9^ cells/mL) was diluted into 6 mL of fresh XT medium to yield a cell density of 5 × 10^3^ cells/mL. The resulting cultures were incubated at 75 °C until they reached OD_600_= 0.1 with shaking. The same procedure was performed for strain HM-8 (OD_600_ = 0.61–0.66, 1.1–1.8 × 10^9^ cells/mL). Each resulting culture was diluted 10^6^–10^8^-fold and spread on XTU plates, and was also spread on XTUF plates without dilution (in triplicate). The plates were incubated at 75 °C for 5 days. The mutation rate was calculated by the numbers of colonies that appeared on the plates.

## 3. Results

### 3.1. Deletion of PolB1-Binding Protein Genes

**Figure 1:**
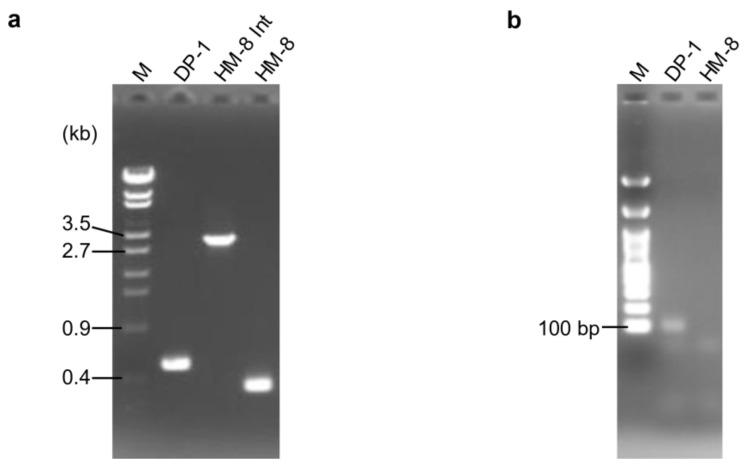
PCR analysis of the *pbp1* gene locus. (**a**) PCR analysis of the *pbp1* locus of the *S. acidocaldarius* DP-1, intermediate (Int), and HM-8 strains using pbp1-out-F/R as primers. The expected sizes of the PCR bands were 0.5 kb (DP-1), 3 kb (HM-8 Int), and 0.4 kb (Δ*pbp1*). A λ-EcoT14 ladder was loaded in lane M. (**b**) PCR analysis of the *pbp1* locus of the *S. acidocaldarius* DP-1 and HM-8 strains using pbp1-in-F/R as primers. The expected sizes of the PCR bands were 87 bp (DP-1) and no band (Δ*pbp1*). A 100-bp DNA ladder was loaded in lane M.

The MONSTER unmarked gene deletion method [23] was applied to the *pbp1* and *pbp2* genes of *S. acidocaldarius*. After transformation, 18 colonies/µg MONSTER-pbp1 were grown. No colony representing the *pbp2* deletion strain could be isolated using either MONSTER-pbp2, which is constructed with an 84 bp deletion, or MONSTER-pbp2n, which is constructed with a 39 bp deletion. One colony showed blue color with X-gal solution was purified and analyzed its genotype by PCR using the outer primers and named strain HM-8 Int (Figure 1a). A total of 8.6 × 10^7^ HM-8 Int cells were then applied for pop-out recombination using XTUF plate, and five white colonies were randomly selected. The genotypes of these colonies exhibited the expected deletion of approximately 0.2 kb in the *pbp1* locus (Figure 1a). We also checked the deletion of the *pbp1* gene using sequencing analysis and confirmed the expected 186 bp deletion in the *pbp1* locus (data not shown). Thus, one correct *pbp1* deletion strain was designated *S. acidocaldarius* strain HM-8 (Δ*pbp1*). In addition, PCR analysis using inner primers yielded no product from HM-8 DNA (Figure 1b), indicating that the *pbp1* gene was deleted from the original genomic locus and was not translocated.

### 3.2. Growth Properties at Various Temperatures

**Figure 2:**
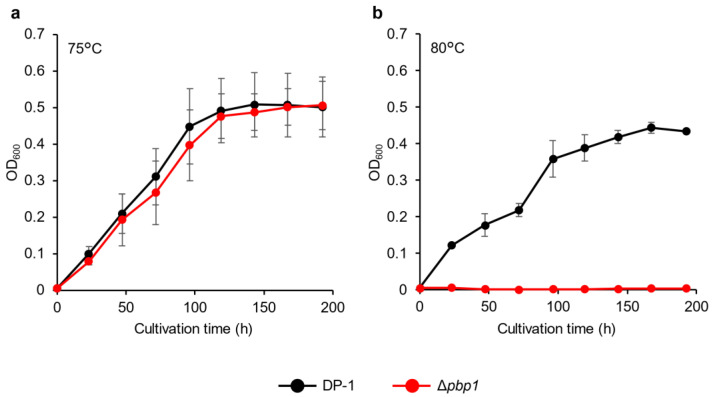
Growth curves of the *pbp1* deletion strain. Overnight cultures of the Δ*pbp1* (HM-8) and DP-1 strains were inoculated into xylose, tryptone, and uracil (XTU) liquid medium and cultivated at 75 °C (**a**) and 80 °C (**b**) without shaking. The error bars indicate the mean ± SD, calculated from triplicate experiments. Black line: The growth of DP-1; red line: The growth of Δ*pbp1* (HM-8).

The growth of deletion strain HM-8 (Δ*pbp1*) was compared to that of the parent strain DP-1 over a wide temperature range (50–80 °C). At 80 °C, no growth of the Δ*pbp1* strain was observed, while the parent strain could grow (Figure 2). Between 50 °C and 75 °C, the growth of the Δ*pbp1* strain was nearly the same as that of the parent strain in the logarithmic growth phase (Figure S1).

### 3.3. Sensitivity to UV Irradiation

**Figure 3:**
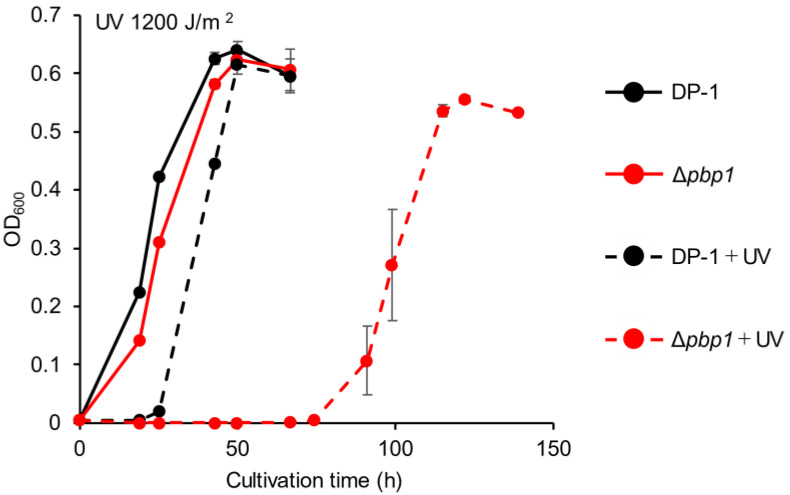
Growth of the *pbp1* deletion strain after UV-B irradiation. Overnight cultures of the Δ*pbp1* (HM-8) and DP-1 strains were irradiated with UV for 60 s (1200 J/m^2^) and cultivated at 75 °C with shaking. +UV represents a UV-treated sample. The error bars indicate the mean ± SD calculated from triplicate experiments. Black line: The growth of DP-1; red line: The growth of Δ*pbp1* (HM-8).

The growth of Δ*pbp1* after UV-B irradiation (zero, 400, 800, 1200, and 1600 J/m^2^) was characterized. The growth curves of Δ*pbp1* and the parent strain without irradiation were nearly the same (Figure 3). After UV irradiation at 400 J/m^2^, slight growth retardation of Δ*pbp1* was observed (Figure S2a). This retardation was clearer after UV irradiation at 800 J/m^2^ (Figure S2b). The difference became more striking after UV irradiation at 1200 (Figure 3) and 1600 J/m^2^ (Figure S2c). The results indicated that Δ*pbp1* exhibited significant sensitivity to helix-distorting lesions such as cyclobutane pyrimidine dimers (CPDs) and pyrimidine (6-4) pyrimidine photoproducts (6-4PP) induced by UV irradiation [28,29,30].

### 3.4. Sensitivity to Chemical Mutagens

**Figure 4:**
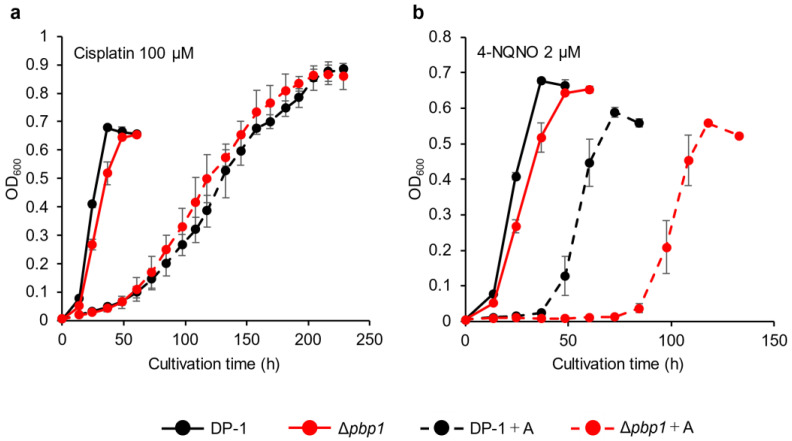
Growth of the *pbp1* deletion strain in the presence of DNA-damaging agents. Overnight cultures of the Δ*pbp1* (HM-8) and DP-1 strains were inoculated into liquid medium in the presence of DNA-damaging agents (cisplatin (100 μM (**a**)) and 4-nitroquinoline N-oxide (4-NQNO) (2 μM (**b**)) and cultivated at 75 °C with shaking. +A represents the growth with DNA-damaging agents. The error bars indicate the mean ± SD, calculated from triplicate experiments. Black line: The growth of DP-1; red line: The growth of Δ*pbp1* (HM-8).

The sensitivity of Δ*pbp1* to other helix-distorting lesions was also tested. Δ*pbp1* and the parent strain were incubated in growth medium with or without cisplatin (Wako, Chuo-Ku, Osaka, Japan) (70 and 100 μM). In the presence of cisplatin, the growth of Δ*pbp1* was the same as that of the parent strain (Figure 4a, Figure S3a). The growth of Δ*pbp1* was also tested in the presence or absence of 4-nitroquinoline N-oxide (4-NQNO) (TCI, Tokyo, Tokyo, Japan) (1 and 2 μM). In the presence of 1 μM 4-NQNO, the growth of Δ*pbp1* was retarded compared with that of the parent strain (Figure S3b). At 2 μM, the difference became more striking (Figure 4b). These results indicated that Δ*pbp1* exhibited significant sensitivity to bulky adducts by induced 4-NQNO, but did not show increased sensitivity to DNA intra strand and inter strand cross-links induced by cisplatin.

**Figure 5:**
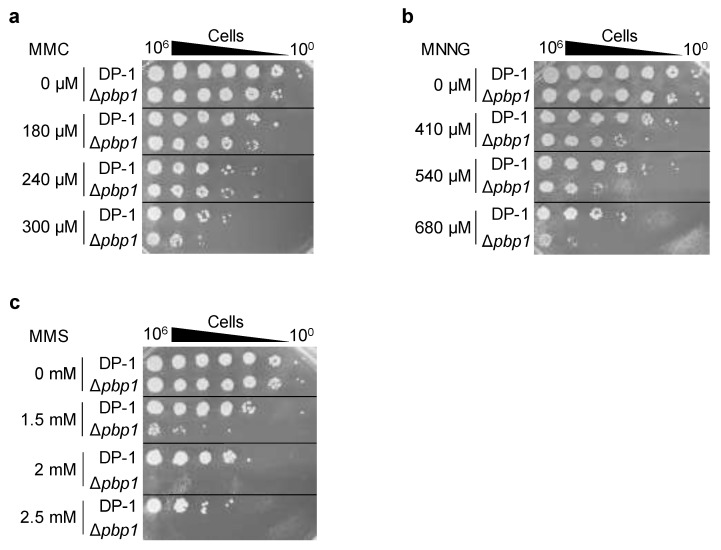
Sensistivity to mitomycin C (MMC), methylnitronitrosoguanidine (MNNG), and methyl methanesulfonate (MMS) of the *pbp1* deletion strain. DP-1 and Δ*pbp1* (HM-8) strains were treated with (**a**) MMC (0, 180, 240, and 300 μM), (**b**) MNNG (0, 410, 540, and 680 μM), and (**c**) MMS (0, 1.5, 2, and 2.5 mM), diluted (10^0^–10^−6^), spotted onto XTU plates and cultivated at 75 °C.

To analyze the sensitivity of Δ*pbp1* to mitomycin C (MMC) (Wako, Chuo-Ku, Osaka, Japan), mock- and MMC-treated (zero, 180, 240, and 300 μM) aliquots of Δ*pbp1* and the parent strain were spotted on plates. No sensitivity of Δ*pbp1* to MMC (180 and 240 μM) was observed (Figure 5a). At 300 μM, the survival of Δ*pbp1* was slightly decreased in comparison with that of the parent strain (Figure 5a). The results suggested that Δ*pbp1* exhibited slight sensitivity to DNA inter strand crosslinks induced by MMC.

To examine additional types of DNA damage, the cells of Δ*pbp1* and the parent strain were treated with methylnitronitrosoguanidine (MNNG) (SIGMA, Kawasaki, Kanagawa, Japan) and methyl methanesulfonate (MMS) (Wako, Chuo-Ku, Osaka, Japan) and were spotted on plates. The survival of Δ*pbp1* treated with MNNG (410 μM) was decreased compared to that of the parent strain, and this difference became more striking at 540 and 680 μM (Figure 5b). The survival of Δ*pbp1* after treatment with MMS (1.5 mM) was dramatically decreased in comparison with that of the parent strain, and this difference also became more striking at 2 and 2.5 mM (Figure 5c). These results indicated that Δ*pbp1* exhibited sensitivity to methylated base induced by MNNG or MMS. In particular, Δ*pbp1* showed greater sensitivity to 7-methylguanine and 3-methyladenine induced by MMS than *O^6^*-methylguanine induced by MNNG [31].

### 3.5. Sensitivity to Heat-Shock Treatment

**Figure 6:**
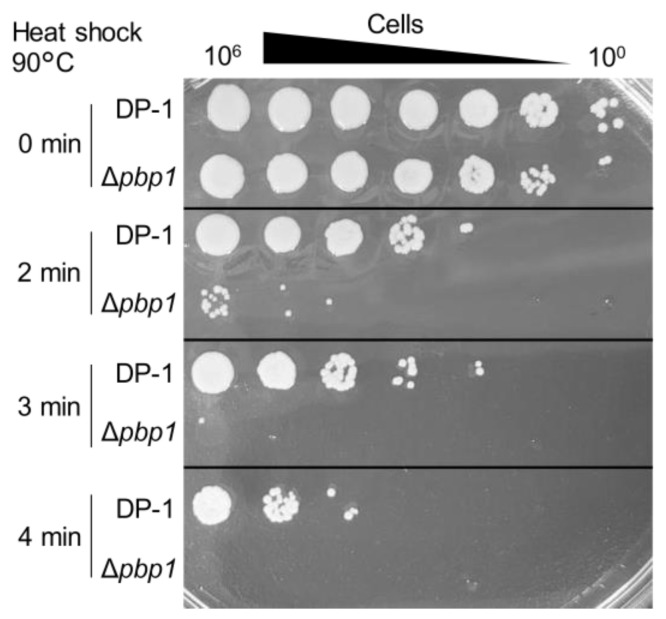
Heat-shock sensitivity of the *pbp1* deletion strain. After heat-shock at 90 °C for 0–4 min, diluted samples (10^−6^–10^0^) of the DP-1 and Δ*pbp1* (HM-8) strains were spotted onto XTU plates and cultivated at 75 °C.

The aliquots of Δ*pbp1* and the parent strain were heated at 90 °C for 0–4 min and spotted onto XTU plates. The survival of Δ*pbp1* was dramatically less than that of the parent strain after 2 min at 90 °C (Figure 6). This difference became more striking at longer heating times (3 or 4 min) (Figure 6). The results indicated that Δ*pbp1* was significantly sensitive to heat shock, which accelerates such reactions as follows (e.g., deamination, methylation, oxidation, and the formation of apurinic/apyrimidinic sites (AP sites)).

### 3.6. Sensitivity to DNA Replication Inhibitors

**Figure 7:**
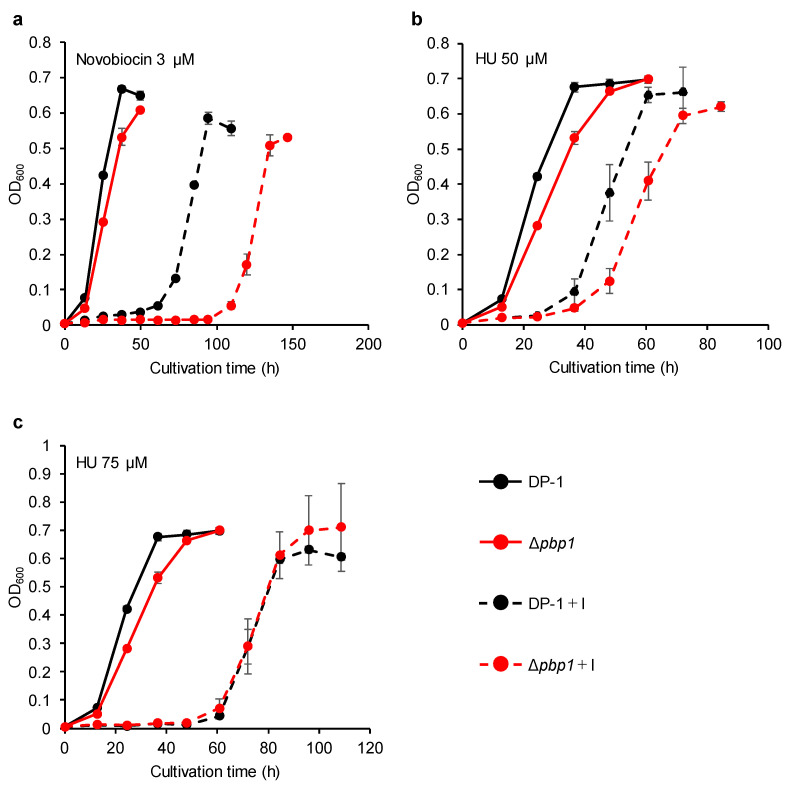
Growth of the *pbp1* deletion strain in the presence of DNA replication inhibitors. Overnight cultures of the Δ*pbp1* (HM-8) and DP-1 strains were inoculated into liquid medium in the presence of a DNA replication inhibitor (novobiocin (3 μM (**a**)) and HU (50 (**b**) and 75 μM (**c**))) and cultivated at 75 °C with shaking. +I represents the growth with a DNA replication inhibitor. The error bars indicate the mean ± SD, calculated from triplicate experiments. Black line: The growth of DP-1; red line: The growth of Δ*pbp1* (HM-8).

The growth of Δ*pbp1* in the presence of novobiocin (1.5 μM) was retarded compared with that of the parent strain (Figure S4a). The difference became more striking in the presence of novobiocin (3 (Figure 7a), 4.5 (Figure S4b), and 6 μM (Figure S4c)). In the presence of HU (25 μM), the growth of Δ*pbp1* was nearly the same as that of the parent strain (Figure S5a). In the presence of HU (50 μM), the growth of Δ*pbp1* was slightly delayed compared to the parent strain (Figure 7b). However, the growth of Δ*pbp1* was the same as that of the parent strain in the presence of HU (75 (Figure 7c) and 100 μM (Figure S5b)). These results indicated that Δ*pbp1* was highly sensitive to novobiocin. Novobiocin, a well-known topoisomerase inhibitors in bacteria and/or eukaryotes, was reported to slow down or arrest chromosome replication at elongation stage in *S. acidocaldarius* [32]. On the other hand, Δ*pbp1* did not exhibit sensitivity to HU in this study in contrast to the chromosome replication that was perturbed in *S. solfataricus* by an unknown mechanism [33].

### 3.7. Estimation of Mutation Rates

We investigated the mutation frequency of Δ*pbp1*. Mutation assays revealed that the mutation rate of Δ*pbp1* was 10-fold higher than that of the parent strain (4.3 (±0.2) ×10^−5^ for the parent strain vs. 3.3 (±0.8) ×10^−4^ for Δ*pbp1*). The results indicated that PBP1 is important for mutation avoidance.

## 4. Discussion

To examine whether PBP1 and PBP2 are essential for the activity of apo-PolB1, we attempted to delete the *pbp1* and *pbp2* genes independently in *S. acidocaldarius*. As a result, a *pbp1* deletion strain was constructed; however, no *pbp2* deletion strain was isolated. These results demonstrated that PBP2 is essential for DNA replication by apo-PolB1. In addition, Δ*pbp1* exhibited sensitivity to numerous types of DNA damage, suggesting that PBP1 is important in DNA repair or the tolerance of DNA damage by apo-PolB1.

PolB1 has been found in all members of the TACK (Thaumarchaota, Aigarchaota, Crenarchaeota, and Korarchaeota) superphylum of Archaea [10,11]. PBP1 and PBP2 are present in the order *Sulfolobales*, *Acidilobales*, and *Desulfurococcales*, which belong to Crenarchaea [11,20]. On the other hand, Thaumarchaea, Aigarchaea, Korarchaea, and Crenarchaea of the order *Thermoproteales* do not possess homologs of PBP1 or PBP2 [11,20]. Thaumarchaea, Aigarchaea, and Korarchaea also possess D-family polymerase, which is a replicative polymerase, in addition to PolB1, while Crenarchaea of the order *Thermoproteales* lack a D-family polymerase [11,20]. Almost all *Thermoproteales* have acidic extensions in the N-terminal regions of PolB1, which may serve as alternatives playing the role of PBP1 [20]. Similarly, the alternatives playing the role of PBP2 may be present in *Thermoproteales* since PBP2 is essential for the activity of PolB1 in *S. acidocaldarius*.

The development and application of PCR technology using thermophilic bacterial and archaeal DNAPs has been considered. B-family polymerases of archaea such as *Pyrococcus furiosus*, *Thermococcus kodakarensis*, and *Thermococcus litralis* are often used as PCR enzymes [34,35,36]. B-family polymerases of *Sulfolobales* have not been practically used for PCR, but attempts have been made to apply them for PCR. The suitability of PolB3, but not PolB1 for PCR, has been verified in Crenarchaea [37,38,39,40]. This may be attributed to the absence of PBP2, which is essential for replication by apo-PolB1. In addition, holoenzyme PolB1 in *S. solfataricus* is capable of performing PCR [20].

In this study, the growth of the Δ*pbp1* strain was nearly the same as that of the parent strain at 75 °C (optimal growth condition), although PBP1 is important for lagging strand DNA synthesis [20]. In addition, Δ*pbp1* exhibited sensitivity to various types of DNA damage, suggesting that PBP1 is involved in DNA repair or damage tolerance rather than lagging strand synthesis by apo-PolB1. A previous in vitro study reported that two chromatin proteins, Sso7d (Sul7d) and Cren7, inhibited the robust strand displacement by apo-PolB1 in *S. solfataricus* [41]. Sul7d is highly conserved in *Sulfolobus* [42], whereas Cren7 (an essential gene in *Sulfolobus islandicus* [43]) is widely conserved in Crenarchaea, except for *Thermophilum pendens* [44]. Taken together, Cren7 and Sul7d, but not PBP1, are mainly responsible for inhibiting excessive strand displacement by apo-PolB1 during Okazaki fragment maturation [41]. DNA repair by apo-PolB1 is possibly enabled by inhibiting excessive displacement of apo-PolB1 during gap filling. Bacteria have PolI, which is an A-family polymerase and is involved in the maturation of the Okazaki fragments at the lagging strand [5]. PolI has 5′ to 3′ exonuclease activity to remove the ribonucleotide portion of newly synthesized Okazaki fragments and DNA polymerase activity to fill in the resulting gap [45]. In addition, PolI fills in DNA gaps that result from the removal of a variety of DNA lesions (e.g., the UV-induced thymidine dimer, the oxidative lesion 8-oxoguanine, and the alkylation lesion 4-methyladenine) during repair [45]. Holoenzyme PolB1 in archaea seems to play the roles of both PolI, which removes RNA primers and fills the gap in DNA repair, and PolIII, which replicates leading and lagging strands in bacteria.

In this study, no growth of Δ*pbp1* was observed at 80°C. Genetic evidence indicates that PBP1 is important for the thermostability of apo-PolB1, consistent with a previous in vitro study showing that holoenzyme PolB1 in the presence of PBP1 and PBP2 causes a large increase in the thermostability of the enzyme compared to apo-PolB1 [20]. Our results showed that Δ*pbp1* exhibited high sensitivity to various types of damage, suggesting that holoenzyme PolB1 contributes to DNA repair or to the tolerance of broad types of DNA damage. In particular, Δ*pbp1* is substantially sensitive to UV irradiation, MMS, 4-NQNO, heat shock, and novobiocin. *S. acidocaldarius* has three accessory DNAPs, namely, PolB2, PolB3, and Dbh. These deletion strains, including double and triple mutants, did not exhibit sensitivity to MMS compared with the parent strain [15]. In addition, these deletion strains were not sensitive to novobiocin at 75 °C [15]. This indicates that holoenzyme PolB1 rather than three accessory DNAPs mainly contributes to the repair or tolerance of damage induced by MMS and novobiocin. A previous in vivo study indicated that the Δ*polB2* Δ*polB3* combination was sensitive to UV, but the effect was limited in magnitude [15]. This study showed that Δ*pbp1* exhibited significant sensitivity to UV irradiation, suggesting that holoenzyme PolB1 is mainly involved in the repair or tolerance of UV damage rather than PolB2, PolB3, and Dbh. The DNA damage induced by heat shock (e.g., deamination, methylation, oxidation, and the formation of AP sites) and methylated base induced by MNNG and MMS are thought to be repaired by base excision repair (BER) or alternative excision repair (AER) [2,3,46]. On the other hand, helix-distorting DNA lesions such as CPDs, 6-4PP, and bulky adducts induced by UV irradiation and 4-NQNO are thought to be repaired by the nucleotide excision repair (NER) [2,3,47]. It is suggested that PBP1 is involved in gap filling by holoenzyme PolB1 in these DNA repair pathways. However, thermophilic archaea are known to lack some NER proteins, so the mechanism by which helix-distorting DNA damage is repaired is interesting, but unknown in archaea [3,47,48,49]. On the other hand, Δ*pbp1* was not sensitive (or was slightly sensitive) to MMC and cisplatin, which induce inter strand DNA crosslinks. In addition, Δ*pbp1* did not exhibit sensitivity to HU, which is an inhibitor of DNA synthesis. Although the mechanism of repair of inter strand cross-linking is not well understood in archaea, PBP1 may not be involved directly in the repair of inter strand cross-linking.

Interaction with PBP1 reduced 3′ to 5′ exonuclease activity compared to that of apo-PolB1 [20]. It was speculated that 3′ to 5′ exonucleolytic proofreading was promoted in the absence of PBP1 in vivo. However, the mutation rate of Δ*pbp1* was significantly increased compared to that of the parent strain. These results suggested that inhibition of the 3′ to 5′ exonuclease activity by PBP1 had no direct influence on accurate replication, but indicated that the effects of proofreading by holoenzyme PolB1 may be complicated. A moderate 3′ to 5′ exonuclease activity is probably necessary for DNA integrity.

## 5. Conclusions

To examine whether PBP1 and PBP2 are essential for the activity of apo-PolB1 in *S. acidocaldarius*, we attempted to delete the *pbp1* and *pbp2* genes independently. It was possible to construct a Δ*pbp1* strain, but not a Δ*pbp2* strain. In addition, Δ*pbp1* exhibited high sensitivity to various types of damage and an increased mutation rate. In particular, Δ*pbp1* exhibited greater sensitivity to UV irradiation, MMS, and novobiocin than the deletion strains of *polB2*, *polB3*, and *dbh,* including double and triple mutants [15]. These results suggested that holoenzyme PolB1 contributes to both replication and repair. PBP1 is involved in the repair or tolerance of various types of DNA damage, although it is not essential for the activity of apo-PolB1. On the other hand, PBP2 is essential for replication by apo-PolB1. Thus, holoenzyme PolB1 of *S. acidocaldarius* is versatile. These results provide new genetic evidence of the biological function of holoenzyme PolB1.

## Figures and Tables

**Table 1:** Strains and DNA sequences used in this study.

Strains or DNAs	Relevant Characteristic(s)	Source or Reference
**Strains**		
*S. acidocaldarius*		
DP-1	SK-1 with Δ*phr* (Δ*pyrE* Δ*suaI* Δ*phr*)	[23,24]
HM-8	DP-1 with Δ*pbp1* (Δ*pyrE* Δ*suaI* Δ*phr* Δ*pbp1*)	This study
**Plasmid DNA**		
placSpyrE	Plasmid DNA carrying 0.8 kb of the 5′ and 3′ homologous regions of the *suaI* locus at both ends of the *pyrE*-*lacS* dual marker	[23]
**PCR products**		
MONSTER-pbp1	Linear DNA containing the 38-bp 5′ and 30-bp 3′ sequences of the *pbp1* flanking regions and a 38-bp region of *pbp1* as the Tg-arm at both ends of the *pyrE*-*lacS* dual marker	This study
MONSTER-pbp2	Linear DNA containing the 38-bp 5′ and 30-bp 3′ sequences of the *pbp2* flanking regions and a 38-bp region of *pbp2* as the Tg-arm at both ends of the *pyrE*-*lacS* dual marker	This study
MONSTER-pbp2n	Linear DNA containing the 38-bp 5′ and 30-bp 3′ sequences of the *pbp2* flanking regions and a 38-bp region of *pbp2* as the Tg-arm at both ends of the *pyrE*-*lacS* dual marker	This study

**Table 2:** Primers used in this study.

Primers	Sequence (5′-3′) ^1^
MONSTER-pbp1-F	tatacgtttcaaaatgcaaatattaaaaatagttagaa**gagcacgtactctcacataatttctcatac**TGTTTTTCTCTATATCAATCTC
MONSTER-pbp1-R	gttttccattttggcgtccaacgtgtagttgatgacatACTCCTAGATCTAAAACTAAAG
MONSTER-pbp2-F	attattatatagtaatggaatttataaggtgaagctta**aaggctcttggaataagtgatccagagaaa**TGTTTTTCTCTATATCAATCTC
MONSTER-pbp2-R	aaatatttcttcgccttctctaattcgtcctctggcaaACTCCTAGATCTAAAACTAAAG
MONSTER-pbp2n-F	aggacgaattagagaaggcgaagaaatatttccagaac**aaggctcttggaataagtgatccagagaaa**TGTTTTTCTCTATATCAATCTC
MONSTER-pbp2n-R	aattccctgaccgctaaaatctcgcctacggaaactacACTCCTAGATCTAAAACTAAAG
pbp1-out-F	tgatgatgacaatttgaatctc
pbp1-out-R	aattcctcctagcatgtatac
pbp1-in-F	aagatatagatatctgttttgac
pbp1-in-R	tttggcgtattaccttttttac
SAMR31-F	gatttcgtgaaagctctacttg
SAMR31-R	tttttctcagctctgatgtatc

^1^ The common sequence for the amplification of the pyrE-lacS dual marker and the 5′, 3′, and Tg regions are indicated by capital letters, underlining, bold font, and double lines, respectively.

## Data Availability

The original contributions presented in the study are included in the article/supplementary material, and further inquiries can be directed to the corresponding author/s.

## Supplementary Materials

The following are available online at https://www.mdpi.com/2076-2607/9/2/439/s1, Figure S1. Growth curves of the pbp1 deletion strain. Figure S2. Growth of the pbp1 deletion strain after UV-B irradiation. Figure S3. Growth of the pbp1 deletion strain in the presence of DNA-damaging agents. Figure S4. Growth of the pbp1 deletion strain in the presence of novobiocin. Figure S5. Growth of the pbp1 deletion strain in the presence of HU.

## References

[1] Johansson E., Dixon N. (2013). Replicative DNA polymerases. Cold Spring Harb. Perspect. Biol..

[2] White M.F., Garrett R.A., Klenk H.P. (2007). DNA repair. Archaea: Evolution, Physiology and Molecular Biology.

[3] White M.F., Allers T. (2018). DNA repair in the archaea–an emerging picture. FEMS Microbiol. Rev..

[4] McHenry C.S. (2011). Bacterial replicases and related polymerases. Curr. Opin. Chem. Biol..

[5] Raia P., Delarue M., Sauguet L. (2019). An updated structural classification of replicative DNA polymerases. Biochem. Soc. Trans..

[6] Kunkel T.A., Burgers P.M. (2008). Dividing the workload at a eukaryotic replication fork. Trends Cell Biol..

[7] Sarmiento F., Long F., Cann I., Whitman W.B. (2014). Diversity of the DNA replication system in the archaea domain. Archaea.

[8] Doublie S., Zahn K.E. (2014). Structural insights into eukaryotic DNA replication. Front. Microbiol..

[9] Jain R., Aggarwal A.K., Rechkoblit O. (2018). Eukaryotic DNA polymerases. Curr. Opin. Struct. Biol..

[10] Makarova K.S., Krupovic M., Koonin E.V. (2014). Evolution of replicative DNA polymerases in archaea and their contributions to the eukaryotic replication machinery. Front. Microbiol..

[11] Cooper C.D.O. (2018). Archaeal DNA polymerases: New frontiers in DNA replication and repair. Emerg. Top. Life Sci..

[12] Cubonová L., Richardson T., Burkhart B.W., Kelman Z., Connolly B.A., Reeve J.N., Santangelo T.J. (2013). Archaeal DNA polymerase D but not DNA polymerase B is required for genome replication in *Thermococcus kodakarensis*. J. Bacteriol..

[13] Sarmiento F., Mrázek J., Whitman W.B. (2013). Genome-scale analysis of gene function in the hydrogenotrophic methanogenic archaeon *Methanococcus maripaludis*. Proc. Natl. Acad. Sci. USA.

[14] Kushida T., Narumi I., Ishino S., Ishino Y., Fujiwara S., Imanaka T., Higashibata H. (2019). Pol B, a family B DNA polymerase, in *Thermococcus kodakarensis* is important for DNA repair, but not DNA replication. Microbes Environ..

[15] Miyabayashi H., Jain R., Suzuki S., Grogan D.W., Kurosawa N. (2020). PolB1 is sufficient for DNA replication and repair under normal growth conditions in the extremely thermophilic crenarchaeon *Sulfolobus acidocaldarius*. Front. Microbiol..

[16] Feng X., Liu X., Xu R., Zhao R., Feng W., Liao J., Han W., She Q. (2020). A unique B-Family DNA polymerase facilitating error-prone DNA damage tolerance in Crenarchaeota. Front. Microbiol..

[17] Cann I.K.O., Komori K., Toh H., Kanai S., Ishino Y. (1998). A heterodimeric DNA polymerase: Evidence that members of Euryarchaeota possess a distinct DNA polymerase. Proc. Natl. Acad. Sci. USA.

[18] Sauguet L., Raia P., Henneke G., Delarue M. (2016). Shared active site architecture between archaeal PolD and multi-subunit RNA polymerases revealed by X-ray crystallography. Nat. Commun..

[19] Klimczak L.J., Grummt F., Burger K.J. (1985). Purification and characterization of DNA polymerase from the archaebacterium *Sulfolobus acidocaldarius*. Nucleic Acids Res..

[20] Yan J., Beattie T.R., Rojas A.L., Schermerhorn K., Gristwood T., Trinidad J.C., Albers S.V., Roversi P., Gardner A.F., Abrescia N.G.A. (2017). Identification and characterization of a heterotrimeric archaeal DNA polymerase holoenzyme. Nat. Commun..

[21] Sakai H.D., Kurosawa N. (2018). *Saccharolobus caldissimus* gen. nov., sp. nov., a facultatively anaerobic iron-reducing hyperthermophilic archaeon isolated from an acidic terrestrial hot spring, and reclassification of *Sulfolobus solfataricus* as *Saccharolobus solfataricus* comb. nov. and *Sulfolobus shibatae* as *Saccharolobus shibatae* comb. nov. Int. J. Syst. Evol. Microbiol..

[22] Cranford M.T., Kaszubowski J.D., Trakselis M.A. (2020). A hand-off of DNA between archaeal polymerases allows high-fidelity replication to resume at a discrete intermediate three bases past 8-oxoguanine. Nucleic Acids Res..

[23] Suzuki S., Kurosawa N. (2017). Development of the multiple gene knockout system with one-step PCR in thermoacidophilic crenarchaeon *Sulfolobus acidocaldarius*. Archaea.

[24] Suzuki S., Kurosawa N. (2016). Disruption of the gene encoding restriction endonuclease *Sua*I and development of a host-vector system for the thermoacidophilic archaeon *Sulfolobus acidocaldarius*. Extremophiles.

[25] Grogan D.W., Robb F.T., Place A.R., Sowers K.R., Schreier H.J., DasSarma S., Fleishmann E.M. (1995). Isolation of *Sulfolobus acidocaldarius* mutants. Archaea: A Laboratory Manual-Thermophiles.

[26] Suzuki S., Kurosawa N. (2019). Endonucleases responsible for DNA repair of helix-distorting DNA lesions in the thermophilic crenarchaeon *Sulfolobus acidocaldarius* in vivo. Extremophiles.

[27] Reilly M.S., Grogan D.W. (2001). Characterization of intragenic recombination in a hyperthermophilic archaeon via conjugational DNA exchange. J. Bacteriol..

[28] Courcelle J., Crowley D.J., Hanawalt P.C. (1999). Recovery of DNA replication in UV-irradiated *Escherichia coli* requires both excision repair and RecF protein function. J. Bacteriol..

[29] Lopes M., Foiani M., Sogo J.M. (2006). Multiple mechanisms control chromosome integrity after replication fork uncoupling and restart at irreparable UV lesions. Mol. Cell.

[30] Dorazi R., Götz D., Munro S., Bernander R., White M.F. (2007). Equal rates of repair of DNA photoproducts in transcribed and non-transcribed strands in *Sulfolobus solfataricus*. Mol. Microbiol..

[31] Lindahl T. (1993). Instability and decay of the primary structure of DNA. Nature.

[32] Hjort K., Bernander R. (2001). Cell cycle regulation in the hyperthermophilic crenarchaeon *Sulfolobus acidocaldarius*. Mol. Microbiol..

[33] Liew L.P., Lim Z.Y., Cohen M., Kong Z., Marjavaara L., Chabes A., Bell S.D. (2016). Hydroxyurea-mediated cytotoxicity without inhibition of ribonucleotide reductase. Cell Rep..

[34] Cariello N.F., Swenberg J.A., Skopek T.R. (1991). Fidelity of *Thermococcus litoralis* DNA polymerase (Vent^TM^) in PCR determined by denaturing gradient gel electrophoresis. Nucleic Acids Res..

[35] Lundberg K.S., Shoemaker D.D., Adams M.W., Short J.M., Sorge J.A., Mathur E.J. (1991). High-fidelity amplification using a thermostable DNA polymerase isolated from *Pyrococcus furiosus*. Gene.

[36] Takagi M., Nishioka M., Kakihara H., Kitabayashi M., Inoue H., Kawakami B., Oka M., Imanaka T. (1997). Characterization of DNA polymerase from *Pyrococcus* sp. strain KOD1 and its application to PCR. Appl. Environ. Microbiol..

[37] Kähler M., Antranikian G. (2000). Cloning and characterization of a family B polymerase from the hyperthermophilic crenarchaeon *Pyrobaculum islandicum*. J. Bacteriol..

[38] Seo K.J., Cho S.S., Ppyun H.W., Youn M.H., Kim S.H., Seo B.S., Kwon S.T. (2014). Characterization of a family B DNA polymerase from the hyperthermophilic crenarchaeon *Ignicoccus hospitalis* KIN4/I and its application to PCR. Appl. Biochem. Biotechnol..

[39] Daimon K., Ishino S., Imai N., Nagumo S., Yamagami T., Matsukawa H., Ishino Y. (2018). Two family B DNA polymerases from *Aeropyrum pernix*, based on revised translational frames. Front. Mol. Biosci..

[40] Iwai T. (2001). Functional and phylogenetic analysis of DNA polymerase and cofactor PCNA in Crenarchaeota. Ph.D. Thesis.

[41] Sun F., Huang L. (2013). *Sulfolobus* chromatin proteins modulate strand displacement by DNA polymerase B1. Nucleic Acids Res..

[42] Choli T., Henning P., Wittmann-Liebold B., Reinhardt R. (1988). Isolation, characterization and microsequence analysis of a small basic methylated DNA-binding protein from the Archaebacterium, *Sulfolobus solfataricus*. Biochim. Biophys. Acta.

[43] Zhang C., Phillips A.P.R., Wipfler R.L., Olsen G.J., Whitaker R.J. (2018). The essential genome of the crenarchaeal model *Sulfolobus islandicus*. Nat. Commun..

[44] Guo L., Feng Y., Zhang Z., Yao H., Luo Y., Wang J., Huang L. (2007). Biochemical and structural characterization of Cren7, a novel chromatin protein conserved among Crenarchaea. Nucleic Acids Res..

[45] Patel P.H., Suzuki M., Adman E., Shinkai A., Loeb L.A. (2001). Prokaryotic DNA polymerase I: Evolution, structure, and “base flipping” mechanism for nucleotide selection. J. Mol. Biol..

[46] Yasui A. (2013). Alternative Excision Repair Pathways. Cold Spring Harb. Perspect. Biol..

[47] Grogan D.W. (2015). Understanding DNA repair in hyperthermophilic archaea: Persistent gaps and other reactions to focus on the fork. Archaea.

[48] Fujikane R., Ishino S., Ishino Y., Forterre P. (2010). Genetic analysis of DNA repair in the hyperthermophilic archaeon, *Thermococcus kodakarensis*. Genes Genet. Syst..

[49] Zhang C., Tian B., Li S., Ao X., Dalgaard K., Gökce S., Liang Y., She Q. (2013). Genetic manipulation in *Sulfolobus islandicus* and functional analysis of DNA repair genes. Biochem. Soc. Trans..

