# Supplementary material for: DNA Polymerase B1 Binding Protein 1 Is Important for DNA Repair by Holoenzyme PolB1 in the Extremely Thermophilic Crenarchaeon Sulfolobus acidocaldarius
Source: Microorganisms. 2021 Feb 20;9(2):439. doi: 10.3390/microorganisms9020439 (PMC7923795; doi:10.3390/microorganisms9020439)
Supplement: Supplementary file 1 [file microorganisms-09-00439-s001.pdf]

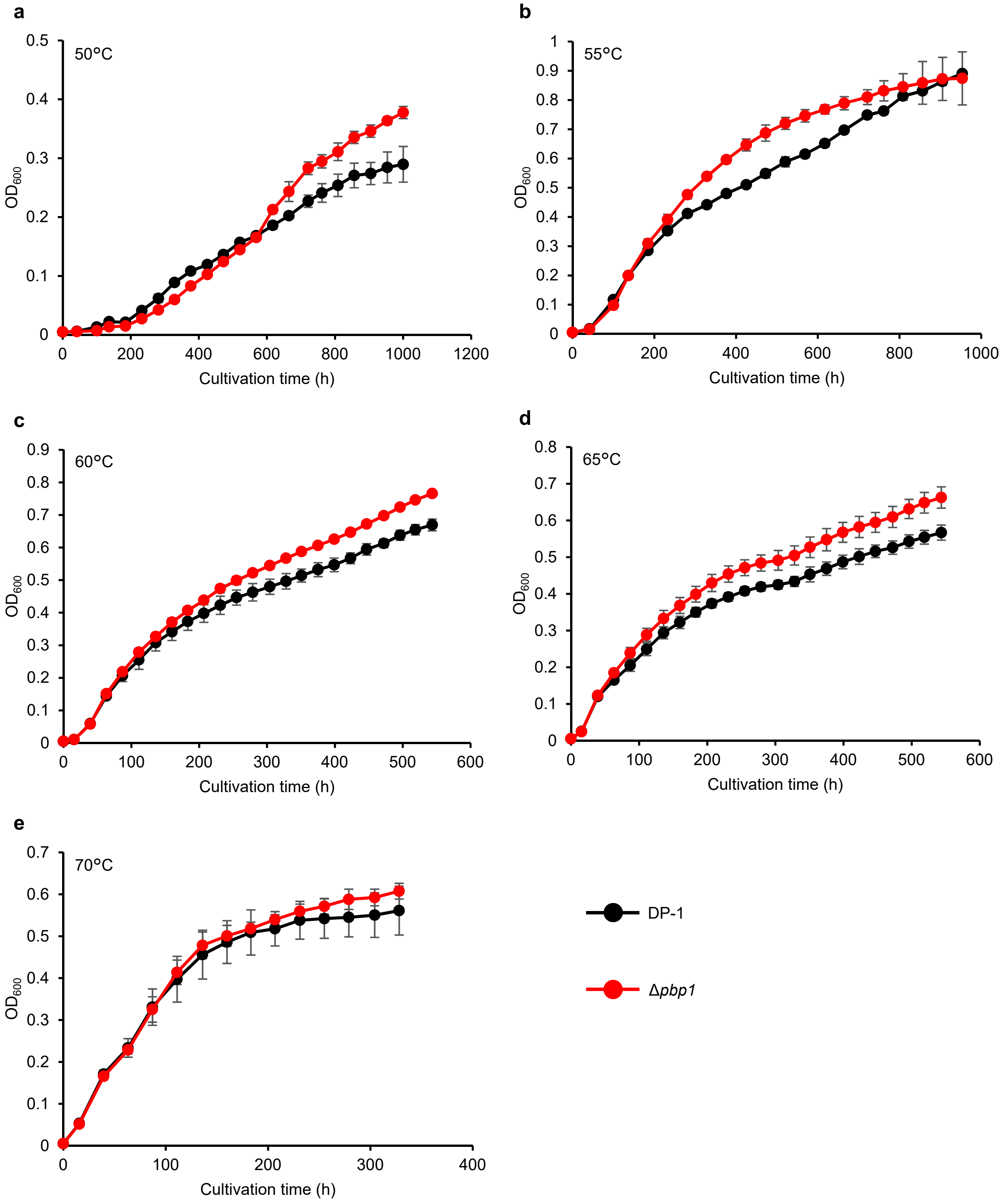

Figure S1. Growth curves of the *pbp1* deletion strain. Overnight cultures of the Δ*pbp1* (HM-8) and DP-1 strains were inoculated into XTU liquid medium and cultivated at 50–70 °C (**a**–**e**) without shaking. The error bars indicate the mean ± SD, calculated from triplicate experiments. Black line: the growth of DP-1; red line: the growth of Δ*pbp1* (HM-8).

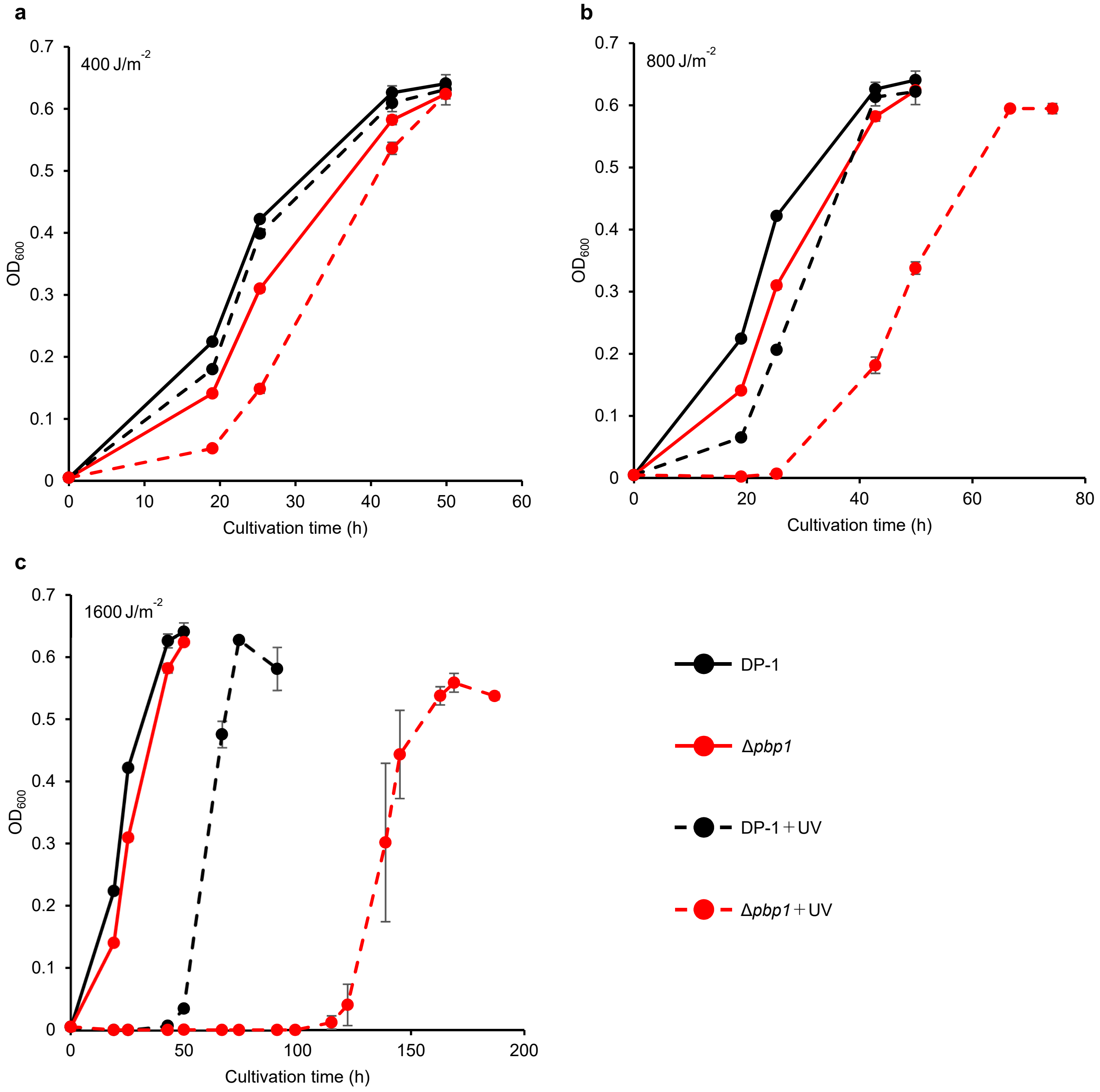

Figure S2. Growth of the *pbp1* deletion strain after UV-B irradiation. Overnight cultures of the Δ*pbp1* (HM-8) and DP-1 strains were irradiated with UV for 20, 40, and 80 sec (400 (**a**), 800 (**b**), and 1600 J/m^2 (**c**)) and cultivated at 75 °C with shaking. +UV represents a UV-treated sample. The error bars indicate the mean ± SD calculated from triplicate experiments. Black line: the growth of DP-1; red line: the growth of Δ*pbp1* (HM-8).

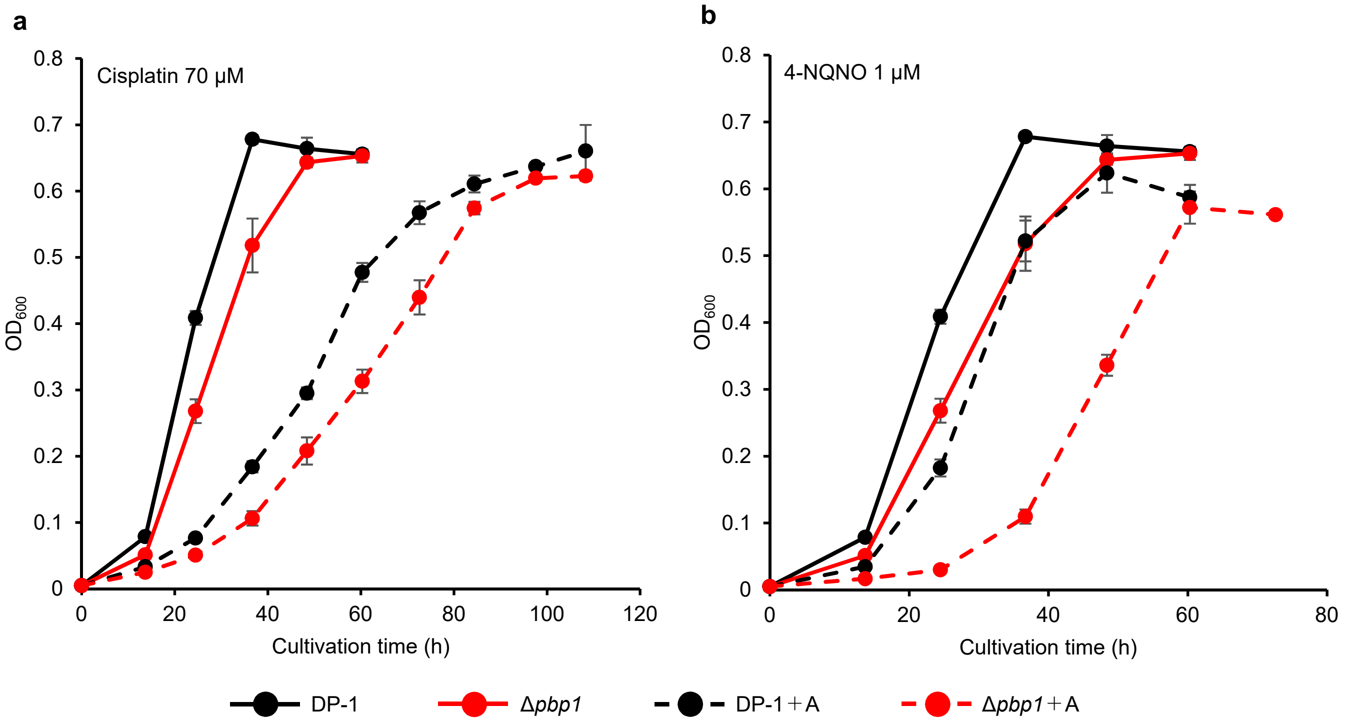

Figure S3. Growth of the *pbp1* deletion strain in the presence of DNA-damaging agents. Overnight cultures of the Δ*pbp1* (HM-8) and DP-1 strains were inoculated into liquid medium in the presence of DNA-damaging agents (cisplatin (70 μM (**a**)) and 4-NQNO (1 μM (**b**)) and cultivated at 75 °C with shaking. +A represents the growth with DNA-damaging agents. The error bars indicate the mean ± SD, calculated from triplicate experiments. Black line: the growth of DP-1; red line: the growth of Δ*pbp1* (HM-8).

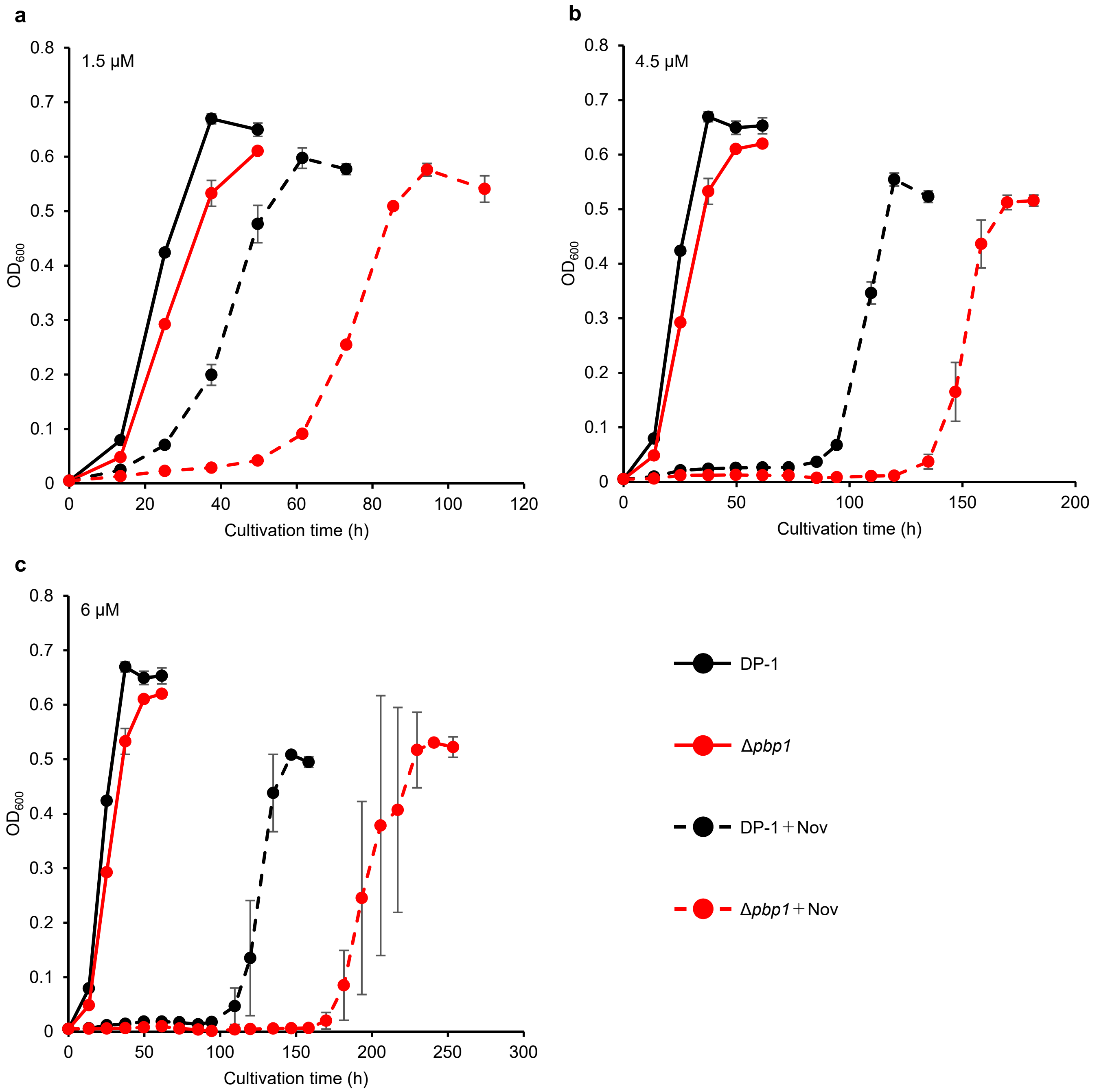

Figure S4. Growth of the *pbp1* deletion strain in the presence of novobiocin. Overnight cultures of the Δ*pbp1* (HM-8) and DP-1 strains were inoculated into liquid medium in the presence of novobiocin (1.5 (**a**), 4.5 (**b**), and 6 μM (**c**)) and cultivated at 75 °C with shaking. +Nov represents the growth with novobiocin. The error bars indicate the mean ± SD, calculated from triplicate experiments. Black line: the growth of DP-1; red line: the growth of Δ*pbp1* (HM-8).

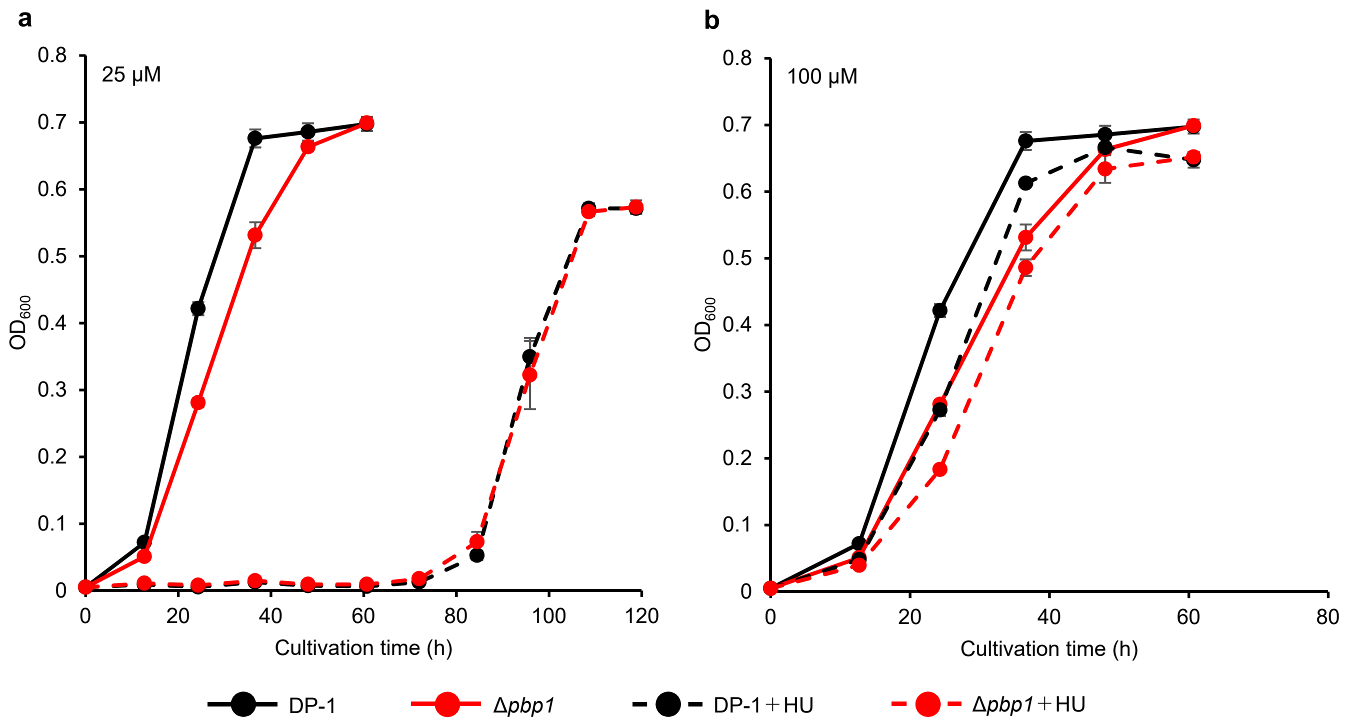

Figure S5. Growth of the *pbp1* deletion strain in the presence of HU. Overnight cultures of the Δ*pbp1* (HM-8) and DP-1 strains were inoculated into liquid medium in the presence of HU (25 (**a**) and 100 μM (**b**)) and cultivated at 75 °C with shaking. +HU represents the growth with HU. The error bars indicate the mean ± SD, calculated from triplicate experiments. Black line: the growth of DP-1; red line: the growth of Δ*pbp1* (HM-8).
